# Tumors of the lateral and third ventricle: surgical management and outcome analysis in 42 cases

**DOI:** 10.17712/nsj.2017.4.20170149

**Published:** 2017-10

**Authors:** Sherif M. Elwatidy, Abdulrahman A. Albakr, Abdullah A. Al Towim, Safdar H. Malik

**Affiliations:** *From the Division of Neurosurgery, Department of Surgery, College of Medicine, King Saud University, Riyadh, Kingdom of Saudi Arabia*

## Abstract

**Objectives:**

To discuss the clinical presentation, pathological diagnosis, and surgical outcome for a series of 42 consecutive patients treated for lateral and third ventricular tumors.

**Methods:**

This is a retrospective series study conducted between 2001 and 2015 and included 42 patients (mean age: 25 years; range: 2 months-65 years) with lateral and third ventricle tumors surgically treated at King Khaled University Hospital, Riyadh, Kingdom of Saudi Arabia. Demographic, clinical, radiological, surgical, histopathological, and follow up data were analyzed.

**Results:**

The most common symptoms at presentation included headache (69%), nausea/vomiting (38%), visual deficits (24%), and seizures (17%). Lesions were located in the lateral ventricle in 15 patients, third ventricle in 20 patients, and involved both the lateral and third ventricles in 7 patients. The most common tumor types in the overall cohort were colloid cysts (n=6) and pineal tumors (n=6). The postoperative complication rate was 36%. The most common postoperative complications were seizure and hydrocephalus (n=5 each, 12%). Surgical mortality was 5%.

**Conclusion:**

The selection of the surgical approach for intraventricular tumor resection is fundamentally dependent on the surgeon’s experience and preference. We recommend that this decision be based on the anatomic considerations that provide the best and safest access to the mass, rather than on the risk of seizure following transcortical approach.

Tumors of the lateral and third ventricles present a unique challenge to the neurosurgeon due to their deep location and relationship with vital neural and vascular structures. Supratentorial and infratentorial intraventricular tumors are commonly observed in children, representing approximately 41% of lateral and third ventricular tumors. However, such tumors account for only 7% of cases in the adult population.^1^,^2^ Approximately half of all adult intraventricular mass lesions are located in the lateral ventricle, whereas the percentage is much lower in children.^1^,^2^ Intraventricular mass lesions include a large variety of benign and malignant tumors.^3^-^8^ In the adult population, the most frequently encountered tumors of the lateral ventricle include astrocytoma, meningioma, glioblastoma, ependymoma, and choroid plexus papilloma.^6^,^9^ In contrast, the most frequently encountered tumors of the lateral ventricle in the pediatric population include subependymal giant cell astrocytoma, choroid plexus papilloma, ependymoma, astrocytoma, and choroid plexus carcinoma.^5^ Surgical resection remains the main method for the treatment of lateral and third ventricular tumors, although intraventricular lesions may be accessed via different surgical approaches. The selection of the surgical approach depends mainly on the location, size, and type of the lesion; the size of the ventricles; the relationship of the tumor to the third ventricle; vascularity; venous drainage; and the relationship of the lesion to surrounding structures.^10^ While transcortical or transcallosal approaches can result in complete tumor resection, such approaches are associated with a significantly increased risk of injury to the brain parenchyma.^11^,^12^ In addition, the morbidity rate for these approaches can reach up to 70%, while mortality ranges from 0-36%.^6^,^7^,^13^ In the present report, we discuss the clinical presentation, pathological diagnosis, and surgical outcome for a series of 42 consecutive patients treated for lateral and third ventricular tumors.

## Methods

We performed a retrospective review of data from all patients undergoing surgery for lateral and third ventricular tumors between 2001 and 2015 in the Department of Neurosurgery at King Khaled University Hospital, Riyadh, Kingdom of Saudi Arabia. We initially identified 47 patients who underwent surgical resection for lateral and third ventricular tumors. Five charts were excluded because of missing data, resulting in a total of 42 eligible patients. We reviewed the medical records, radiological images, pathology reports, and surgical reports of each patient. We also collected data regarding demographic information, preoperative signs and symptoms, surgical approach, tumor location on imaging, histopathological diagnosis, extent of surgical resection, and postoperative complications.

Preoperative signs and symptoms, as well as postoperative complications, were determined from clinical charts. Pathological diagnosis was confirmed postoperatively. One to 3 months following surgery, all patients underwent radiologic workup including brain computed tomography (CT) and/or magnetic resonance imaging (MRI) with gadolinium contrast to assess the extent of surgical resection. Computed tomography scans were performed within 48 hours after surgery to exclude the presence of postoperative complications such as hydrocephalus and hemorrhage. The protocol of the present study was approved by the institutional review board of our hospital prior to initiation of the study. Descriptive statistics were performed for the variables and expressed as (frequencies, mean, and range).

## Results

The study series included 42 patients (24 adults and 18 children; 20 male/22 female) with lateral and third ventricular tumors. The mean patient age at surgery was 25 years (range: 2 months to 65 years). Mean age in the adult population was 34 years (range: 20 to 65 years), while mean age in the pediatric population was 10 years (range: 2 months to 18 years).

The most common symptoms at presentation in the overall series included headache 29 (69%), nausea/vomiting 16 (38%), visual deficits 10 (24%), seizures 7 (17%), change in behavior 6 (14%), unstable gait 4 (10%), altered mental status 4 (10%), motor deficits 3 (7%), and dizziness 2 (5%). In the adult population, headache 83%, nausea/vomiting 33%, and visual deficits 33% were observed. The most common symptoms in the pediatric population included headache 50% and nausea/vomiting 44%, while seizures were reported in 28% of patients. The preoperative signs and symptoms of adult and pediatric patients are summarized in **Table 1**.

**Table 1:** Clinical features of patients with lateral and third ventricular tumors.

Signs and symptoms	Pediatrics	Adults	All patients
	n (%)		
Headache	9 (50)	20 (83)	29 (69)
Nausea-vomiting	8 (44)	8 (33)	16 (38)
Visual deficit*	2 (11)	8 (33)	10 (24)
Seizures	5 (28)	2 (8)	7 (17)
Change in behavior	2 (11)	4 (17)	6 (14)
Unstable gait*	3 (17)	1 (4)	4 (10)
Altered mental status	1 (6)	3 (13)	4 (10)
Motor deficit*	-	3 (13)	3 (7)
Dizziness	-	2 (8)	2 (5)
Urine incontinence	2 (11)	-	2 (5)
Fever	1 (6)	1 (4)	2 (5)
Tremor	1 (6)	1 (4)	2 (5)
Neck stiffness	1 (6)	-	1 (5)
Tinnitus	-	1 (4)	1 (2)
Endocrine dysfunction	-	1 (4)	1 (2)

* Some patients experienced more than one symptom

Lesions were located in the lateral ventricles in 15 patients, third ventricle in 20 patients, and involved both the lateral and third ventricles in 7 patients. The most common tumor types in the overall cohort were colloid cysts (n=6) and pineal tumors (n=6). The most commonly encountered pathology in the adult population was colloid cyst, while pineal region tumors were more frequently observed in the pediatric population. The histopathological results are summarized in **Table 2**. The postoperative complication rate was 36%. Overall, the most common postoperative complications were seizure and hydrocephalus (n=5 each, 12%). Wound site infection and intraventricular hemorrhage occurred in 3 (7%) and 2 (5%) patients, respectively. Additional complications included weakness, vision disturbance, diabetes insipidus, brain abscess/ventriculitis, and hypothalamic syndrome (n=1 each, 2%). Surgical mortality was reported in 2 patients, resulting from a postoperative brain abscess and ventriculitis in one patient and hypothalamic syndrome in another. Postoperative complications and mortality are summarized in **Table 3**.

**Table 2:** Histopathological types of lateral and third ventricular tumors.

Histopathology	Pediatrics	Adults	All patients
	n (%)		
Pineal region tumors	4 (10)	2 (5)	6 (14)
Colloid cyst	-	6 (14)	6 (14)
Pilocytic astrocytoma	3 (10)	2 (5)	5 (12)
Central neurocytoma	1 (2)	4 (10)	5 (12)
Glioblastoma	1 (2)	3 (7)	4 (10)
Atypical teratoid rhabdoid tumor	3 (7)	-	3 (7)
Ependymoma	-	2 (5)	2 (5)
Meningioma	-	2 (5)	2 (5)
Lymphoma	-	1 (2)	1 (2)
Choroid plexus papilloma	1 (2)	-	1 (2)
Anaplastic ependymoma	1 (2)	-	1 (2)
Anaplastic astrocytoma	1 (2)	-	1 (2)
Mature teratoma	1 (2)	-	1 (2)
Anaplastic oligodendroglioma	-	1 (2)	1 (2)
Craniopharyngioma	-	1 (2)	1 (2)
Glioneuronal tumor	1 (2)	-	1 (2)
Langerhans cell histiocytosis	1 (2)	-	1 (2)
**Total**	**18**	**24**	**42**

**Table 3:** Postoperative complications and mortality in patients with lateral and third ventricular tumors

Complications	all patients n (%)
Seizure	5 (12)
Hydrocephalus	5 (12)
Wound site infection	3 (7)
Intraventricular hemorrhage	2 (5)
Weakness	1 (2)
Vision disturbance	1 (2)
Diabetes insipidus	1 (2)
Mortality	2 (5)
Hypothalamic syndrome	1 (2)
Brain abscess and Ventriculitis	1 (2)

*Some patients experienced more than one complication

**Figure 1:**
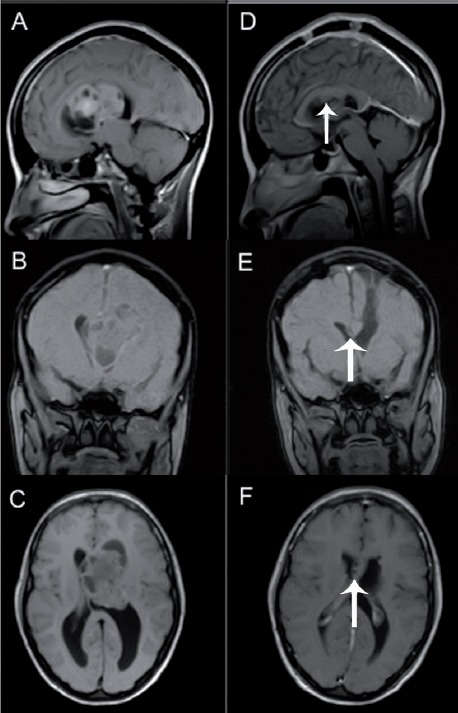
A 30-year-old woman presenting with a headache, blurry vision, and attacks of generalized body numbness for 2 months. **A-C)** Sagittal, coronal, and axial T1 Magnetic Resonance Imaging (MRI) with and without contrast enhancement showing third ventricular tumor with extension to the left lateral ventricle. She underwent left frontal craniotomy, frontal transcortical approach with subtotal excision of the tumor. The pathology revealed central neurocytoma. **D-F)** Postoperative with and without contrast enhancement T1 sagittal, coronal, and axial scans showing small residual tumor (arrow) under the corpus callosum. She postoperatively developed right side hemiparesis. In her follow-up, 6 and 9 months following surgery, her weakness improved significantly. We think the reason for her weakness is due to the retraction on the motor control areas.

**Figure 2:**
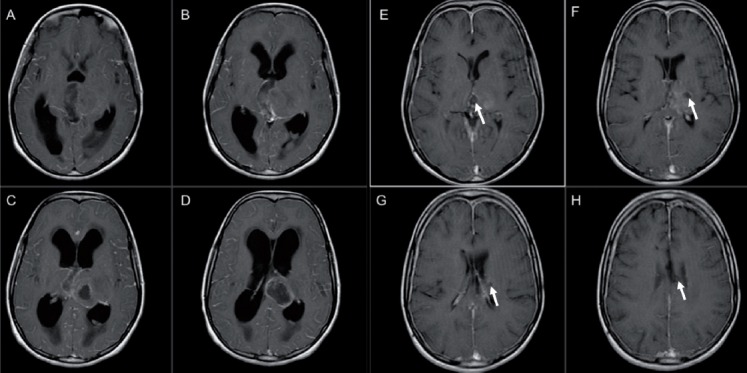
A 10-year-old girl presenting with 2 months history of a persistent headache, vomiting, and tremor in both hands. Additionally, she had 2 episodes of seizures. **A-D)** Contrast-enhanced T1 axial scans showing a mass occupying the left thalamus, crossing the midline, and infiltrating the third ventricle. She underwent resection via transcallosal approach with subtotal excision of the tumor. The pathology revealed central glioblastoma multiforme. **E-H)** Postoperative contrast-enhanced T1 axial scans showing small residual tumor. Postoperatively, the patient recovered very well with no neurological deficits.

All patients underwent surgical resection or biopsy as the initial treatment modality. Gross total resection (GTR) was desired in all cases, except those in which surgical biopsy was intended. However, GTR is considered dangerous and has been associated with high mortality and morbidity in many cases, especially when the lesion is related to critical and vital neurovascular structures. Therefore, subtotal resection (STR) was planned in some patients (**Figure 1 and 2**). Eight patients (19%) underwent endoscopic biopsy. The transcortical approach was utilized in 19 cases (45%), while the transcallosal approach was utilized in five cases. Alternative approaches were utilized in the remaining cases. A total of 34 patients underwent surgical resection. GTR was achieved in 24 (71%) of 34 cases, while STR was performed in the remaining 10 cases (29%). The surgical approaches for the present series are summarized in **Table 4**.

**Table 4:** Surgical approaches in patients with lateral and third ventricular tumors.

Surgical approaches	No. of all patients n (%)
Transcortical approach	19 (45 %)
Endoscopic biopsy	8 (19 %)
Transcallosal approach	5 (12 %)
Infratentorial supracerebellar approach	3 (7 %)
Pterional approach	3 (7 %)
Endoscopic resection	2 (5%)
Posterior fossa craniotomy	1 (2 %)
Subfrontal approach	1 (2 %)

## Discussion

### Clinical presentation

The clinical presentation of lateral and third ventricular tumors varies based on patient age, tumor size, and tumor location. Patients typically present with signs and symptoms secondary to the expansion of the localized mass within the ventricular cavity, such as compression, invasion, edema of periventricular structures, obstruction of the normal cerebrospinal fluid (CSF) flow, and hydrocephalus. While obstruction of CSF flow may result in hydrocephalus, overproduction of CSF by the tumor represents another underlying mechanism. The mass effect that results in hydrocephalus often impairs the fornices and leads to short-term memory loss. Less frequently, intraventricular mass lesions may bleed, resulting in acute deterioration in clinical status. Because of the variable location of intraventricular tumors, however, no stereotypic neurologic or behavioral signs or symptoms can be expected in such disease.

The most common signs and symptoms at presentation are related to increased intracranial pressure (ICP) and include headache, nausea, emesis, and somnolence.^2^,^9^,^14^ These and other signs of elevated ICP were present in most patients in the current series. In children, the most common clinical presentation of lateral ventricle tumors includes headache, disturbance in gait, cognitive disturbance, and visual problems. Additionally, an increase in head circumference is commonly noted in infants.^5^ In one of the largest series of patients with lateral ventricular tumors (n=112), Gokalp et al^9^ observed papilloedema in 42.9% of patients, headache in 35.7%, motor disturbance in 25%, sensory disturbance in 25%, and nausea/vomiting in 22.3%. In another study that examined the clinical presentation of patients with tumors of the third ventricle, headache was noted as the chief complaint in 26 patients (68%), while memory deficits were observed in 45%, visual deficits were observed in 32%, and gait disturbances were observed in 21% of patients, respectively.^7^ Milligan et al^13^ reported that, among 127 patients with lesions in the lateral and third ventricles, signs and symptoms related to high ICP were noted in 85%, cognitive difficulties in 44%, weakness in 33%, seizures in 13%, and visual changes in 17%.

In our series, the clinical presentations were similar in adult and pediatric patients, with the most common symptoms at presentation related to increased ICP. However, the rate of seizures was higher in children 28% than adults 8%, although visual deficits were more frequent among adult patients (33% vs. 11%). Weakness and unstable gait usually result from the edema/compression induced by increased ICP following tumor invasion into the centrum semiovale, thalamus, basal ganglia, or internal capsule. In the current series, 10% of patients presented with unstable gait, whereas motor deficit was noted in 7%. Our findings are comparable to those reported in previous series.^2^,^9^ Furthermore, previous studies reported that preoperative seizures may occur in 3-13% of patients with intraventricular masses.^2^,^7^,^9^ although we observed preoperative seizure in 17% of our cases.

### Pathology

Intraventricular tumors are rare entities of diverse pathology, representing only 1.3-3% of all intracranial tumors,^2^,^9^,^15^ though they are more commonly observed in children than adults.^15^ The predominant neoplasms of lateral and third ventricular lesions vary significantly with age. Choroid plexus papillomas and malignant small blue cell tumors are commonly observed in the pediatric age group. In contrast, pilocytic astrocytoma, subependymal giant cell astrocytoma, and diffuse low-grade astrocytoma occur mostly in patients aged 6-30 years. Meningiomas, metastasis, and high-grade gliomas are usually observed in patients over 30 years of age.^15^ In a series of 112 patients with lateral ventricular tumors, the most common pathologies were ependymoma (25%), astrocytoma (21.4%), and oligodendroglioma (7.1%).^9^ In another series of 72 patients with lateral ventricle tumors, the most frequently encountered tumors were anaplastic astrocytoma and glioblastoma (24%), meningioma (11%), and ependymoma (7%).^6^ In a series of 127 patients with lateral and third ventricle tumors, Milligan et al^13^ reported that the most common lesions included colloid cysts (27%), pilocytic astrocytomas (14%), and meningiomas (9%). In a series of 54 pediatric patients with lateral ventricle tumors, Zuccaro et al^5^ reported that the most frequent histological types were subependymal giant cell astrocytoma, choroid plexus papilloma, ependymoma, and astrocytoma. In the current series, the most common tumors were pineal region tumors 6 (14%), colloid cysts 6 (14%), and pilocytic astrocytomas 5 (12%). Pineal region tumors were most common in children, whereas colloid cysts were most common in adults.

### Postoperative complications

Potential postoperative complications result from sacrifice of or injury to the structures surrounding the ventricles, deep periventricular ependymal vasculature, or superficial vasculature, as well as cortical retraction when forming the operative corridor. The published rates of postoperative complications for lateral and third ventricle tumors vary widely from one series to another, depending on the definition of complications and timing. However, rates of up to 70% have been reported.^6^,^7^,^13^ Such complications are mainly related to corpus callosum incision by the transcallosal approach, and to white matter injury associated with the cortical incision.

In the current series, the total rate of complications within the first few days after surgery was 36%, in accordance with the findings of previous studies. Most authors have reported postoperative mortality rates between 0% and 36%.^2^,^5^-^7^,^9^,^13^,^15^ In a review of reports from 1935 to 1990, Apuzzo and Litofsky noted that the mortality rate for the transcortical approach was higher than that for the transcallosal approach (13% vs. 6%).^16^ In the present series, we observed postoperative mortality in 2 patients (5%), which compares favorably with these values. Surgical mortality resulted from brain abscess and ventriculitis in one patient and hypothalamic syndrome in another.

### Infection

The risk of ventriculitis should be considered during intraventricular surgery involving the use of external ventricular drains (EVD). In a previous series, meningitis/ventriculitis was reported in 2 patients (5%) who underwent surgery for tumors of the third ventricle, resulting in patient death in one case.^7^ In the present series, only one patient developed brain abscess and ventriculitis, though this patient later died of systemic complications related to sepsis. To minimize the risk of ventriculitis, we utilize the following protocol: (a) tunneling of the EVD catheter as far from the wound site as possible; (b) use of antibiotics within the EVD catheter; (c) use of prophylactic intrathecal antibiotic (three doses of vancomycin or gentamycin [5 mg] 3 days prior to EVD removal) with drain closure for 2 hours following antibiotic administration; (d) shortened period of EVD usage; (e) avoidance of CSF leak around the EVD exit wound via careful titration of EVD level.

### Postoperative seizure

Transcortical approaches for intraventricular tumors are associated with an increased risk of postoperative seizures, with an incidence ranging from 26% to 70% in previous reports.^15^-^17^ However, more recent studies have reported a lower risk of postoperative seizure following transcortical surgery. Ellenbogen et al^18^ reported postoperative seizures in 7% of patients who underwent surgery via the transcortical approach, suggesting that the risk of seizure following transcortical procedures had decreased over time. Similarly, Hassaneen et al^7^ reported that the rate of postoperative seizures was only 3% in 38 patients with tumors of the third ventricle, all of whom have undergone transcallosal procedures. One recent study further reported that the transcallosal approach was associated with significantly increased risk of postoperative seizure when compared with the transcortical approach (8% vs. 25%).^13^ In the current series, only 5 patients (12%) experienced postoperative seizures. Three patients (16%) experienced seizure following transcortical procedures, while no incidence of postoperative seizure was noted following the transcallosal approach. However, as only five patients underwent transcallosal procedures, it is difficult to draw definitive conclusions when comparing these 2 approaches.

### Weakness

Postoperative motor weakness is noted in 8% to 30% of patients undergoing intraventricular tumor resection.^19^ Such complications are presumably the result of retraction and manipulation of motor control areas that are directly involved with or compressed by the tumor. Hemiparesis, focal weakness, and gait instability often result from tumor involvement of the internal capsule, caudate, and thalamus. Importantly, excessive cortical retraction at the premotor cortex and supplementary motor area in transcortical approach (especially if the craniotomy is posterior to the coronal suture) often leads to postoperative weakness. To avoid such issues, we perform the craniotomy 1/3 anterior and 2/3 posterior to the coronal suture.

Retraction at the mesial aspect of the supplementary motor areas during transcallosal procedures may also result in transient weakness. Other causes of weakness include sacrificing of large cortical and bridging veins in the posterior transcollosal approach. Such issues can be alleviated by altering the corridor to preserve the large posterior bridging veins. Other causes of weakness include damage to deep venous structures (e.g., internal cerebral vein) or venous drainage. Hassaneen et al^7^ reported a 5% rate of postoperative weakness in a series of 38 patients who underwent resection via the transcallosal approach. Another study that compared both transcortical and transcallosal approaches revealed similar rates of weakness for transcortical approaches 12% vs. 13% for transcallosal approaches.^13^ In addition, patients who underwent surgery for third ventricular colloid cyst experienced a significantly lower rate of postoperative weakness.^13^ In the current series, persistent weakness was noted in one patient (2%) (**Figure 1**), although 7% of patients presented with weakness prior to surgery.

### Transcallosal Versus Transcortical Approach

The anterior and posterior transcallosal approaches allow for access to and visualization of both lateral ventricles, less disruption to the cerebral cortex, and improved setting of normal ventricular size.^15^ Minimal disruption to the cerebral cortex is one of the main advantages of this procedure, resulting in a lower incidence of postoperative seizures. However, in one recent study by Milligan et al,^13^ this conventional concept was not supported by their results: The transcallosal approach was associated with a significantly increased risk of postoperative seizure. The anterior transcallosal approach provides bilateral access to the anterior horn and bodies of the lateral ventricles, in addition to the anterior third ventricle via the choroidal fissure or interforniceal approach. For the anterior transcallosal approach, safe retraction of the mesial frontal lobe is necessary, although this often results in limited visualization of the lateral and superior aspects of the lateral ventricle. The same limitations can apply to the posterior transcallosal approach, in addition to the risk of weakness from primary motor cortex retraction. Veins bridging to the superior sagittal sinus may limit the access of the transcallosal route. Division of the bridging veins anterior to the coronal suture has been suggested,^15^,^20^ although this procedure has been associated with an increased risk of venous infarction.^21^

The anterior transcortical approach is greatly facilitated by the presence of hydrocephalus or partial obstructions leading to the enlargement of the ventricle, as these provide additional lateral-to-medial access to the frontal horn and body of the ipsilateral lateral ventricle. Transcortical exposure also provides better access to large tumors than a transcallosal route.^2^ Fenestration of the septum pellucidum provides access to the contralateral lateral ventricle. However, difficulties in exposing the contralateral lateral ventricle remain a major limitation of this approach, along with the higher incidence of postoperative seizures.^15^ Extending the approach to include transforaminal and transchoroidal routes may improve access to the third ventricle.

The temporal transcortical approach through the posterior portion of the middle temporal gyrus is selected by many surgeons for the resection of choroidal tumors.^15^ Small lesions primarily located in the temporal horn can be accessed through the inferior temporal gyrus,^15^ as this approach provides a short trajectory to the lesion. Visual field loss and speech disturbance, especially when approaching the lesion through the dominant hemisphere, remain a major limitation of this approach.^22^,^23^ The parietal transcortical approach is often used to access the trigonal region or posterior body of the lateral ventricle. The incidence of language impairment is much lower for this approach than for the temporal transcortical approach.^15^ Previous research suggests that the cortical incision should be made as far as possible from the optic radiations that run lateral and inferior to the trigonal region, in order to reduce the incidence of permanent visual field impairment.^15^ Additionally, apraxia and acalculia or complete Gerstmann syndrome can arise when approaching the lesion through the dominant hemisphere.^24^ Complete resection is often the goal of intraventricular tumors, although the rates of this vary widely in the literature from 38-87%.^6^,^7^,^9^,^13^,^25^ In the present study, GTR was achieved in 71% of the patients, which is similar to some reports but different than in others, perhaps this due to the differences in study composition as regards to tumor size and type.

There are limitations to this study, including its retrospective nature and small study population. Unlike the transcortical approach, the patients who underwent the transcallosal approach were few and therefore making a direct comparison between these 2 approaches in regard to the outcome problematic. In addition, a portion of patients was either lost to follow-up or was followed in different institution with no follow-up information. Accordingly, the assessment of long-term symptomatic outcome was not feasible.

In conclusion, intraventricular tumors remain a challenging entity for neurosurgeons, as such tumors often present with no typical neurological or behavioral signs or symptoms, which may allow the tumor to grow unchecked until more severe manifestations arise. Since they may grow to a considerable size before becoming symptomatic, intraventricular tumors are primarily treated via surgical resection. The selection of the surgical approach is fundamentally dependent on the surgeon’s experience and preference. We recommend that this decision be based on the anatomic considerations that provide the best and safest access to the mass with minimal retraction and risk to neural tissue, rather than on the risk of seizure following transcortical approach.
